# Different prognostic effect of CpG island methylation according to sex in colorectal cancer patients treated with adjuvant FOLFOX

**DOI:** 10.1186/s13148-015-0106-0

**Published:** 2015-07-09

**Authors:** Dae-Won Lee, Sae-Won Han, Yongjun Cha, Ye Young Rhee, Jeong Mo Bae, Nam-Yun Cho, Kyung-Hun Lee, Tae-Yong Kim, Do-Youn Oh, Seock-Ah Im, Yung-Jue Bang, Seung-Yong Jeong, Kyu Joo Park, Gyeong Hoon Kang, Tae-You Kim

**Affiliations:** Department of Internal Medicine, Seoul National University Hospital, 101 Daehang-Ro, Jongno-Gu, Seoul 110-744 South Korea; Cancer Research Institute, Seoul National University College of Medicine, Seoul, Korea; Department of Pathology, Seoul National University College of Medicine, 101 Daehang-Ro, Jongno-Gu, Seoul 110-744 South Korea; Department of Surgery, Seoul National University Hospital, Seoul, Korea; Department of Molecular Medicine and Biopharmaceutical Sciences, Graduate School of Convergence Science and Technology, Seoul National University, Seoul, Korea

**Keywords:** Colorectal cancer, CpG islands methylator phenotype, Sex, FOLFOX

## Abstract

**Background:**

Profound methylation of CpG islands constitutes a distinct molecular subtype of colorectal cancer (CRC). The frequencies of methylation in CRC vary according to clinico-pathological characteristics including sex. However, interaction between these characteristics and prognostic influence of methylation status has not been clearly defined. We have investigated the prognostic role of promoter methylation using eight CpG island methylator phenotype (CIMP) markers in 497 stage II or III CRC patients who underwent curative resection followed by adjuvant FOLFOX. Overall survival (OS) and disease-free survival (DFS) were compared between subgroups classified by methylation status, and interactions with clinico-pathological features were analyzed.

**Results:**

CIMP-high (≥5 methylated loci) and concurrent methylation in *NEUROG1* and *CDKN2A (p16)* were found in 5.8 and 7.9 % of patients, respectively. Although CIMP-high status was not associated with survival, concurrent methylation in *NEUROG1* and *CDKN2A (p16)* was associated with shorter OS and DFS. Moreover, the prognostic role of the concurrent methylation was different among sex. The negative prognostic impact was only observed in male but not in female (interaction *p* value = 0.026 for OS and 0.011 for DFS). In male, the 5-year OS was 61.6 % in concurrent methylation (+) and 91.7 % in concurrent methylation (−) (*p* < 0.001) whereas it was 95.0 and 92.8 % in female, respectively (*p* = 0.78).

**Conclusions:**

Concurrent methylation in *NEUROG1* and *CDKN2A* is associated with poor survival in CRC treated with adjuvant FOLFOX. Interaction analysis indicates that the prognostic role is different according to sex.

## Background

Colorectal cancer develops through various types of genetic and epigenetic alterations, and several critical genes and pathways underlying the carcinogenesis have been elucidated. Most notably, at least three distinct pathways have generally been accepted, the chromosomal instability (CIN), microsatellite instability (MSI), and CpG island methylator phenotype (CIMP) pathways. CIMP is characterized by a high frequency of methylation in numerous promoter CpG islands. CIMP-positive cancers have distinct features compared to CIN cancers that they are more frequently observed in proximal location, older and female patients, and have poor pathologic differentiation [1–3].

There have been controversies in the prognostic role of CIMP in colorectal cancer patients [3–8]. The inconsistency may be related to CIMP definition using different methylation markers and confounding role of other molecular alterations such as MSI or *BRAF* mutation. The prognostic implication of CIMP could also be different according to tumor locations [8, 9]. Moreover, methylation status of individual genes may be more important than the number of methylated markers in determining prognosis. We have recently reported that concurrent methylation in *NEUROG1* and *CDKN2A (p16)* is associated with higher recurrence in colorectal cancer patients whereas CIMP classification based on the number of methylated markers was not [4]. Importance of individual gene methylation such as *CHFR*, *MGMT*, and *SHISA3* has also been shown in other studies [6, 10, 11].

Sex influences clinico-pathological characteristics of colorectal cancer. Male has a higher age-adjusted colorectal cancer incidence and death rate compared to female [12, 13]. The proportion of proximal cancer is higher in female whereas distal colon and rectal cancer is more frequent in male [14]. CIMP also has sexual difference that the frequency is higher in female [1]. The etiology for the sex difference remains uncertain while hormonal factor, dietary factor, and lifestyle factor have been suggested as the cause [15–17].

In the present study, we have analyzed the impact of methylation status on survival in 497 stage III or high-risk stage II colorectal cancer patients treated with adjuvant FOLFOX chemotherapy. We have further investigated whether the prognostic implication is different according to clinico-pathological characteristics including sex.

## Result

### Patients’ characteristics

A total of 497 patients were included in the present study. Baseline characteristics are summarized in Table 1. Tumor location was cecum in 18, ascending colon in 113, transverse in 39, descending in 31, sigmoid in 264, and rectum in 32 patients. Collectively, 169 patients had tumor in proximal (from cecum to transverse colon) location and 328 patients had tumor in distal location. Tumor stage was stage II in 74 patients (IIA in 49, IIB in 21, and IIC in 4) and stage III in 423 patients (IIIA in 39, IIIB in 273, and IIIC in 111). All stage II patients had high-risk features. Microsatellite instability (MSI-high) was shown in 6.5 % of tumors. According to the inclusion criteria, all patients received at least 6 cycles of chemotherapy and 89.9 % of patients completed planned 12 cycles of chemotherapy.

**Table 1:** Baseline characteristics

	Total	CIMP-negative	CIMP-low	CIMP-high	*p* value*
	*N* (%)	*N* (%)	*N* (%)	*N* (%)	
Total	497 (100)	316 (63.6)	152 (30.6)	29 (5.8)	
Age					
<65 years	348 (70.0)	222 (70.3)	108 (71.1)	18 (62.1)	0.34
≥65 years	149 (30.0)	94 (29.7)	44 (28.9)	11 (37.9)	
Sex					
Male	301 (60.6)	201 (63.6)	88 (57.9)	12 (41.4)	0.029
Female	196 (39.4)	115 (36.4)	64 (42.1)	17 (58.6)	
Location					
Proximal	169 (34.0)	87 (27.5)	61 (40.1)	21 (72.4)	<0.001
Distal	328 (66.0)	229 (72.5)	91 (59.9)	8 (27.6)	
BMI					
<25 kg/m^2^	323 (65.0)	197 (62.3)	103 (67.8)	23 (79.9)	0.096
≥25 kg/m^2^	174 (35.0)	119 (37.7)	49 (32.2)	6 (20.7)	
T stage					
T1–3	424 (85.3)	274 (86.7)	128 (84.2)	22 (75.9)	0.14
T4	73 (14.7)	42 (13.3)	24 (15.8)	7 (24.1)	
N stage					
N0–1	364 (73.2)	228 (72.2)	116 (76.3)	20 (69.0)	0.59
N2	133 (26.8)	88 (27.8)	36 (23.7)	9 (31.0)	
Tumor stage					
II, high-risk	74 (14.9)	48 (15.2)	21 (13.8)	5 (17.2)	0.71
III	423 (85.1)	268 (84.8)	131 (86.2)	24 (82.8)	
Histology					
MAC	25 (5.0)	7 (2.2)	12 (7.9)	6 (20.7)	<0.001
Non-MAC	472 (95.0)	309 (97.8)	140 (92,1)	23 (79.3)	
Microsatellite status (*N* = 495)					
MSS/MSI-L	463 (93.5)	306 (97.5)	138 (90.8)	19 (65.5)	<0.001
MSI-H	32 (6.5)	8 (2.5)	14 (9.2)	10 (34.5)	
*KRAS* mutation (*N* = 383)					
Wild type	280 (73.1)	185 (76.4)	77 (64.2)	18 (85.7)	0.18
Mutation	103 (26.9)	57 (23.6)	43 (35.8)	3 (14.3)	
*BRAF* mutation (*N* = 423)					
Wild type	407 (96.2)	270 (99.3)	122 (93.8)	15 (71.4)	<0.001
Mutation	16 (3.8)	2 (0.7)	8 (6.2)	6 (28.6)	

*N* number, *MAC* mucinous adenocarcinoma, *MSS* microsatellite stable, *MSI-L* microsatellite instability-low, *MSI-H* microsatellite instability-high

*p values comparing CIMP-high vs*.* CIMP-low/CIMP-negative by chi-square test

### Methylation status

Methylation at one or more loci was observed in 181 patients (36.4 %, Table 2). *CRABP1* was the most frequently methylated locus, followed by *NEUROG1* and *CDKN2A (p16)*. Twenty-nine patients (5.8 %) had tumors with five or more methylated loci (CIMP-high), 152 patients (30.6 %) had one to four methylated loci (CIMP-low), and 316 patients (63.6 %) had no methylated locus (CIMP-negative). Patients with CIMP-high tumors were designated the CIMP(+), and those with CIMP-low or CIMP-negative tumors were designated the CIMP(−). Patients with following characteristics had higher incidence of CIMP(+): female sex, proximal tumor location, mucinous adenocarcinoma histology, MSI-high, and *BRAF* mutation (Table 1). CIMP(+) tumors had a tendency of lower incidence in obese patients (BMI >25 kg/m^2^ for Asian) compared to CIMP(−) (*p* = 0.096). Incidence of CIMP(+) and individual gene methylation was similar among age.

**Table 2:** Summary of methylation status

Number of methylated loci	Number of patients (%)	Methylation locus	Number of patients with methylation (%)
0	316 (63.6)	CACNA1G	41 (8.2)
1	94 (18.9)	CRABP1	98 (19.7)
2	37 (7.4)	IGF2	30 (6.0)
3	13 (2.6)	MLH1	18 (3.6)
4	8 (1.6)	NEUROG1	87 (17.5)
5	13 (2.6)	CDKN2A (p16)	85 (17.1)
6	9 (1.8)	RUNX3	29 (5.8)
7	4 (0.8)	SOCS1	22 (4.4)
8	3 (0.6)		

We previously reported that concurrent methylation in *NEUROG1* and *CDKN2A (p16)* was associated with higher recurrence [4]. Concurrent methylation in *NEUROG1* and *CDKN2A (p16)* was found in 39 patients (7.9 %). Similar to CIMP(+), concurrent methylation had higher incidence in patients with proximal tumor location, mucinous adenocarcinoma histology, MSI-high, and *BRAF* mutation. Although statistically not significant, concurrent methylation had a tendency of higher incidence in patients with female sex (51.3 vs*.* 38.4 %, *p* = 0.115). In addition, N2 stage was higher in patients with concurrent methylation in *NEUROG1* and *CDKN2A (p16)* (41.0 vs*.* 25.5 %, *p* = 0.036). Incidence of obesity was similar regardless of the concurrent methylation status (25.6 % in patients with concurrent methylation vs*.* 35.8 % in patients without concurrent methylation, *p* = 0.20).

### Prognosis according to methylation status

**Fig. 1:**
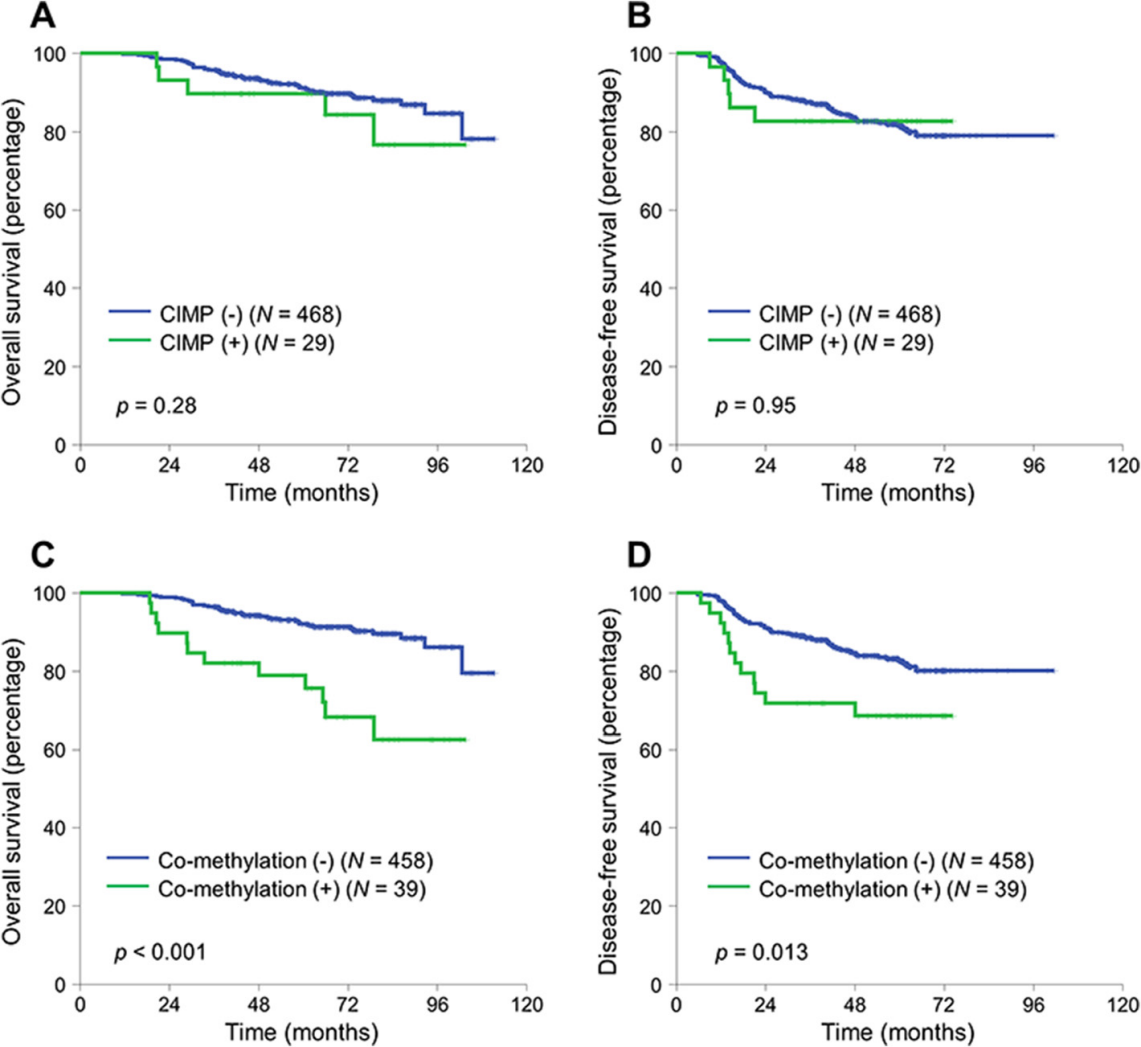
Kaplan-Meier curves of overall survival and disease-free survival according to CIMP status (**a**, **b**) and concurrent methylation of *NEUROG1/CDKN2A (p16)* (**c**, **d**). *Co-methylation* concurrent methylation, *N* number

After a median follow-up duration of 65 months, the 5-year overall survival (OS) of the entire cohort was 91.1 % and the 3-year disease-free survival (DFS) was 87.2 %. There was no significant difference in OS or DFS according to the CIMP status: the 5-year OS was 89.7 % in the CIMP(+) and 91.1 % in the CIMP(−) (*p* = 0.28, Fig. 1a) (DFS, Fig. 1b). There was no difference in the pattern of recurrence (local recurrence vs. distant metastasis) according to the CIMP status.

We next evaluated the influence of concurrent methylation in *NEUROG1* and *CDKN2A (p16)* on survival. Concurrent methylation in *NEUROG1* and *CDKN2A (p16)* was associated with poor OS and DFS (Fig. 1c) (DFS, Fig. 1d). The 5-year OS was 78.9 % in patients with concurrent methylation in *NEUROG1*/*CDKN2A (p16)* and 92.1 % in patients without concurrent methylation in *NEUROG1*/*CDKN2A* (*p* < 0.001). Multivariate analysis using the Cox proportional hazard model revealed that concurrent methylation in *NEUROG1* and *CDK2NA (p16)* was an independent negative prognostic factor for OS (adjusted hazard ratio (HR) for OS 2.89, 95 % confidence interval (CI) 1.45–5.76, *p* = 0.002) but not for DFS.

### Interaction between methylation status and clinico-pathological factors

**Fig. 2:**
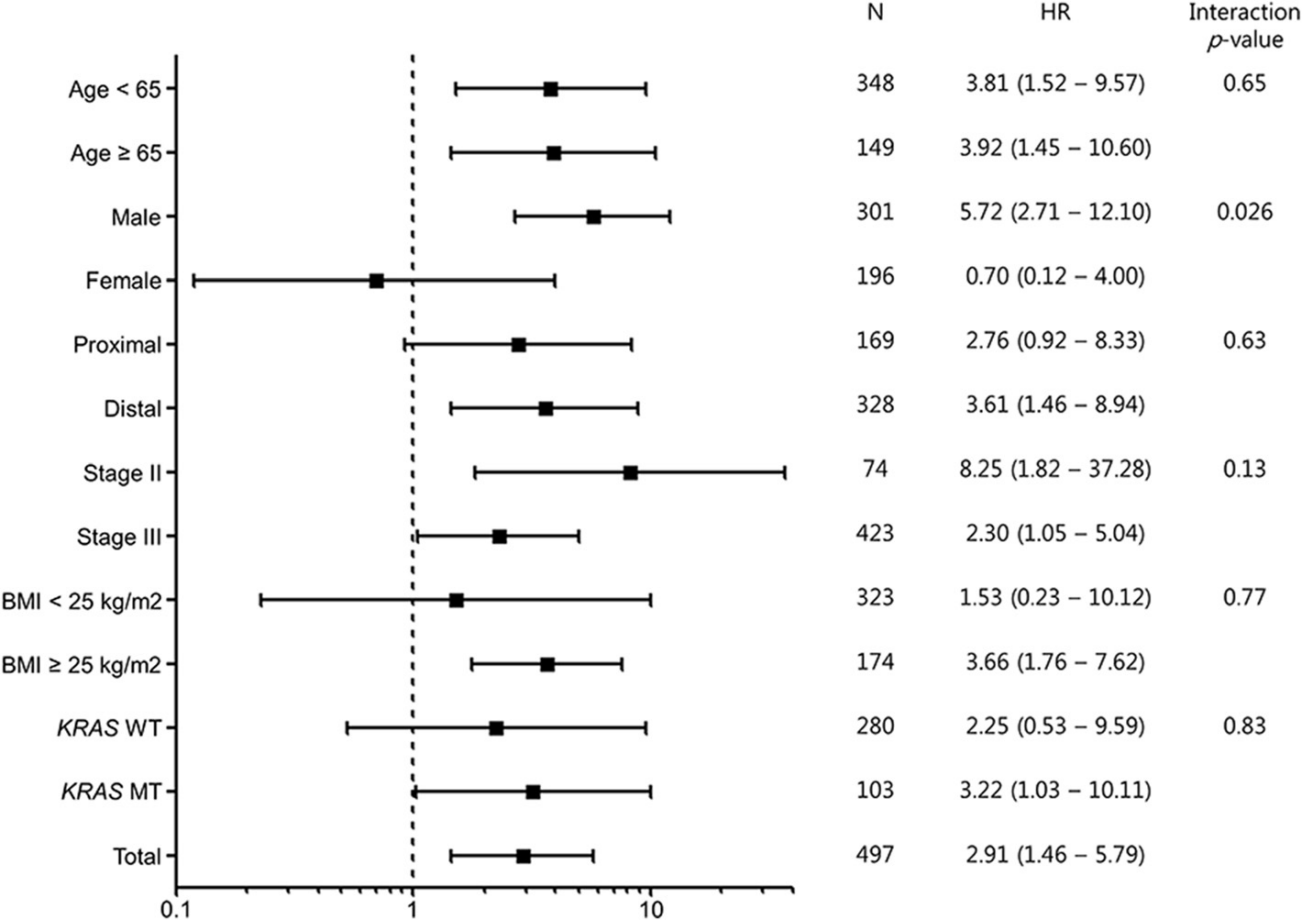
Forest plot demonstrating the risk of death by concurrent methylation (+) compared to concurrent methylation (−) stratified by clinico-pathological factors. All hazard ratios were adjusted by histology, angiolymphatic invasion, and perineural invasion. *N* number, *HR* hazard ratio, *CI* confidence interval, *WT* wild type, *MT* mutation type

**Fig. 3:**
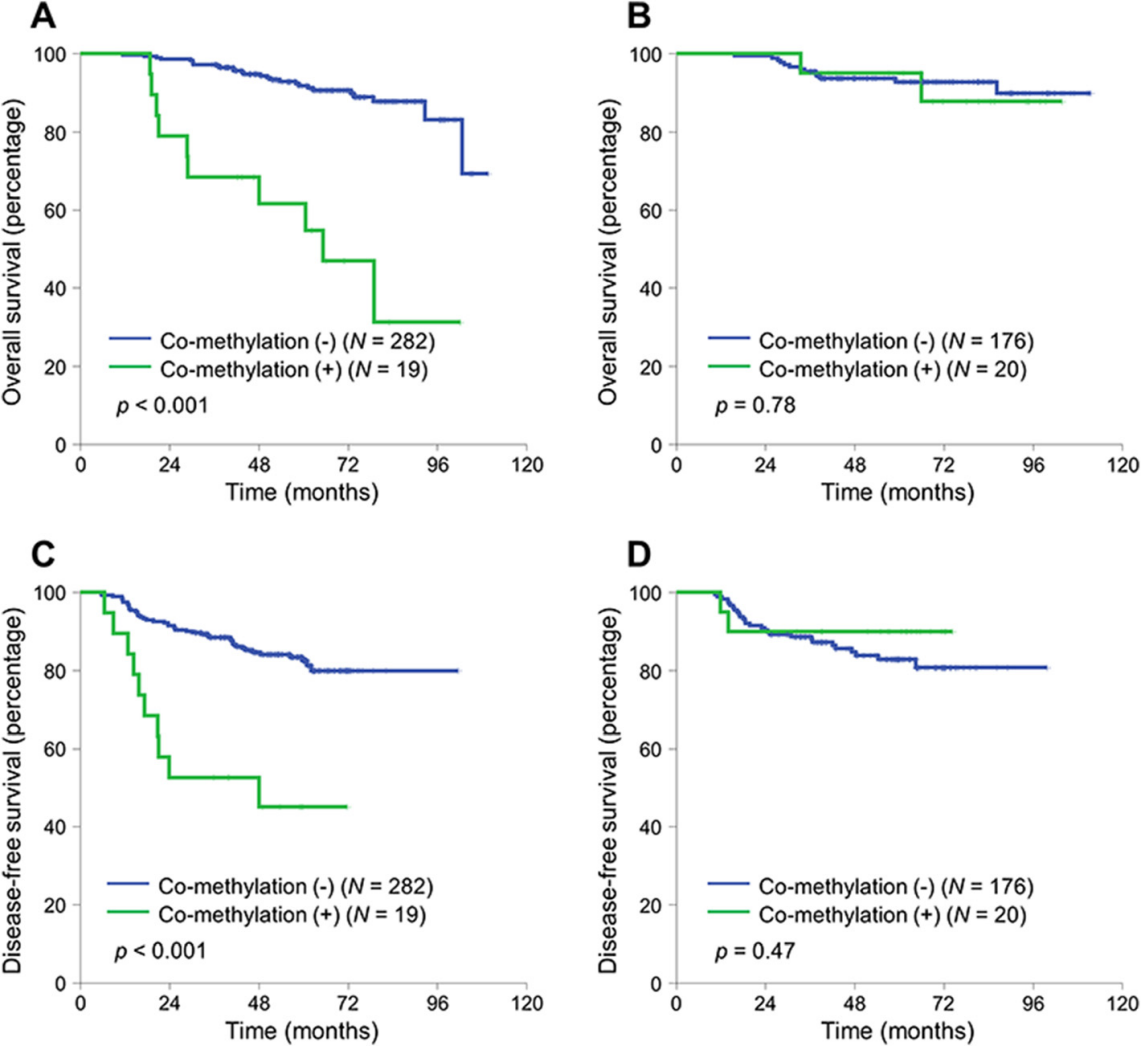
Kaplan-Meier curves of overall survival (OS) and disease-free survival (DFS) according to concurrent methylation of *NEUROG1/CDKN2A (p16)* stratified by sex. (**a**) Male: OS. (**b**) Female: OS. (**c**) Male: DFS. (**d**) Female: DFS. *Co-methylation* concurrent methylation, *N* number

We next assessed whether the detrimental effect of concurrent methylation in *NEUROG1* and *CDKN2A (p16)* was different according to clinico-pathological factors, including sex (Fig. 2). The prognostic role of concurrent methylation in *NEUROG1* and *CDKN2A (p16)* was different among sex (interaction *p* value for OS = 0.026, for DFS = 0.011). It was associated with significantly worse OS and DFS in men (Fig. 3a) (DFS, Fig. 3c). However, there was no prognostic role of concurrent methylation in women (Fig. 3b) (DFS, Fig. 3d). In the multivariate analysis, the poor prognosis associated with concurrent methylation in *NEUROG1* and *CDKN2A (p16)* in male was independent of other clinico-pathologic prognostic factors (adjusted HR for OS 5.23, 95 % CI 2.45–11.17, *p* < 0.001) (adjusted HR for DFS 3.66, 95 % CI 1.82–7.36, *p* < 0.001) (Table 3). Other clinico-pathological factors, including tumor location, did not affect the prognostic role of concurrent methylation in *NEUROG1* and *CDKN2A (p16)*. Due to the limited number of patients, we could not sub-analyze patients according to *BRAF* mutation or MSI status.

**Table 3:** Multivariate analysis of overall survival and disease-free survival among male patients (*N* = 301)

		Overall survival		Disease-free survival	
		Adjusted HR (95 % CI)	*p* value	Adjusted HR (95 % CI)	*p* value
Concurrent methylation	Present	5.23 (2.45–11.17)	<0.001	3.66 (1.82–7.36)	<0.001
	Not present	1		1	
Angiolymphatic invasion	Present	4.41 (1.97–9.85)	<0.001	2.33 (1.30–4.18)	0.005
	Not present	1		1	
Age (continuous variable)		1.032 (0.99–1.07)	0.11		
Histology	MAC	3.47 (0.97–12.41)	0.056		
	Non-MAC	1			
Perineural invasion	Present			2.20 (1.26–3.85)	0.006
	Not present			1	

*CI* confidence interval, *MAC* mucinous adenocarcinoma

## Discussion

In the present study, we have investigated the impact of promoter methylation on treatment outcome of colorectal cancer patients receiving adjuvant FOLFOX chemotherapy. As all patients in our cohort were Korean, our data shows relatively low incidence of MSI-high (MSI-H) and CIMP-high. Previous studies using standardized methodologies have repeatedly shown that the Western shows higher incidence of MSI-H and CIMP-high compared to the Eastern [18]. We observed that CIMP-high is not associated with survival, but concurrent methylation in *NEUROG1* and *CDKN2A (p16)* has deleterious effect in terms of OS and DFS. In addition, the prognostic role of concurrent methylation in *NEUROG1* and *CDKN2A* was different among sex; it was a negative prognostic factor in men but not in women (interaction *p* value of 0.026 for OS and 0.011 for DFS).

Although the prognostic role of CIMP has been extensively investigated in colorectal cancer patients, previous studies have yielded inconsistent results. In the study by Min et al., CIMP-high was a positive prognostic factor in stage II or III colorectal cancer patients treated with fluoropyrimidine-based adjuvant chemotherapy [7]. However, CIMP-high was a negative prognostic factor in stage III, proximal colon cancer patients [8]. These inconsistent results may have attributed from the heterogeneous cohort of patients included in the study, different CIMP-high definitions among studies and confounding role of other molecular characteristics (MSI-high, *KRAS* mutation, and *BRAF* mutation). In addition, there are evidences that each methylation locus differs in their association with survival and clinico-pathological characteristics. Concurrent promoter methylation in *NEUROG1* and *CDKN2A (p16)* was associated with poor DFS in stages II and III colorectal cancer patients, and *CHFR* promoter methylation indicated poor prognosis in stage II colorectal cancer patients [4, 6]. In contrast, considerable proportion of MSI-H in sporadic colorectal cancer results from the loss of *MLH1* expression by methylation of its promoter, and it is known that MSI-H is associated with better prognosis [19, 20]. In our study, CIMP-high was not associated with survival but concurrent methylation in *NEUROG1* and *CDKN2A (p16)* was associated with poor survival. *NEUROG1* is a transcription factor involved in neuronal development and differentiation, and *CDKN2A (p16)* is a tumor suppressor that inhibits cyclin-dependent kinases CDK4 and CDK6 [21, 22]. Although the functional role of promoter methylation in *NEUROG1* and *CDKN2A (p16)* is inconclusive, *CDKN2A (p16)* promoter methylation was associated with poor survival in stages II and III colorectal cancer patients who received adjuvant fluoropyrimidine-based chemotherapy [23]. Classifying CIMP according to the number of methylated loci may be useful for grouping patients with similar clinico-pathological characteristics; however, each promoter methylation may have different prognostic roles, and thus, identifying each methylation locus may be useful in the practice.

There are sex differences in colorectal cancer incidence, death rate, and clinico-pathological characteristics [24]. Female colorectal cancer patients tend to have CIMP-high and proximal tumor location compared to male patients. To our knowledge, no study has revealed the interaction between sex and prognostic impact of promoter methylation yet. In this study, we found that the prognostic role of concurrent methylation in *NEUROG1* and *CDKN2A (p16)* was influenced by sex. It was associated with poor prognosis only in male patients. Previous studies have shown that a negative prognostic role of obesity has sex-related differences [15, 25]. However, we could not find association between methylation status and obesity. Because of the retrospective nature of this study, we could not examine other potential mechanism underlying the sex differences including hormonal factor, dietary factor, and lifestyle factor. Future prospective cohort study may provide answers to the differences.

Other limitation of this study is that only patients treated with adjuvant FOLFOX were included. Therefore, we cannot answer whether the poor prognosis of patients with concurrent methylation in *NEUROG1* and *CDKN2A (p16)* is due to its innate biology or its resistance to adjuvant chemotherapy regimen. However, the major strength of the study is that the study cohort was homogenous, that all patients underwent surgery at a high-volume center and received the same adjuvant FOLFOX chemotherapy, which is the current standard care in patients with stage III colorectal cancer [26]. Our findings need further validation in an independent cohort of patients.

## Conclusions

While CIMP is well known for its role in colon cancer tumorigenesis, the prognostic role of CIMP has not been well defined. In this study, CIMP-high did not have a prognostic role; however, concurrent methylation in *NEUROG1* and *CDKN2A (p16)* was independently associated with poor survival in colorectal cancer patients treated with adjuvant FOLFOX. In addition, the prognostic role of concurrent methylation in *NEUROG1* and *CDKN2A (p16)* was influenced by sex. A negative prognostic role of promoter methylation was shown in men but not in women. Elucidating the underlying mechanism that results in sex difference is warranted in the future.

## Methods

### Patients and adjuvant chemotherapy

This study included 497 pathologically proven stage III or high-risk stage II colorectal cancer patients who received curative surgery followed by adjuvant FOLFOX chemotherapy at Seoul National University Hospital (SNUH; Seoul, Korea) between April 2005 and December 2011. Main inclusion criteria for the retrospective patient selection were age over 18, adenocarcinoma histology, stage III or high-risk stage II, complete resection of the tumor with negative margin, completion of at least 6 cycles of adjuvant FOLFOX chemotherapy. High-risk stage II was defined if the patient had any of the following: T4 lesion, obstruction or perforation, lymphovascular invasion, perineural invasion, or poorly differentiated histology [27]. Patients with upper rectal cancer were included if the patient did not receive pre- or post-operative radiation. Patients were excluded if they met the following criteria: previous chemotherapy for colorectal cancer (CRC), previous radiotherapy for CRC, signet ring cell histology, distant metastasis, and history of other malignancy within 5 years. None of the patients received anti-*EGFR* or anti-*VEGF* treatment adjunct to FOLFOX. Patient received FOLFOX chemotherapy as either FOLFOX-4 (288 patients) or modified FOLFOX-6 (209 patients) regimen [28]. Adjuvant chemotherapy was planned for a total of 12 cycles.

Patients were assessed every 2 weeks during chemotherapy treatment and then at least every 6 months for 5 years. The post chemotherapy period assessment included a medical history taking, physical examination, measurement of the carcinoembryonic antigen level, chest computed tomography, and abdominal computed tomography. The diagnosis of recurrence was made on the basis of imaging and, if necessary, biopsy.

Eligible patients were identified from electronic database, and chart review was performed using the electronic medical record system of SNUH. The study protocol was reviewed and approved by the institutional review board of SNUH.

### Molecular pathologic analysis

Analysis of DNA methylation and microsatellites was performed as previously described [4, 29]. All patients (*N* = 322) included in the previous report were included in the present study [4]. DNA methylation analysis was re-performed for these patients concurrently with the new patients (*N* = 175). In brief, tumor tissue slides were reviewed and areas of high tumor cell density (~1 cm^2^) were marked and dissected with a knife blade. Non-neoplastic colon mucosa tissues were also dissected. The dissected tumor tissues were collected into a microtube containing tissue lysis buffer and proteinase K. Manual microdissection was performed to enrich tumor cell DNA proportion in the sample DNA, because the results of quantitative MethyLight analysis may be influenced by high proportions of contaminating normal cells. After sodium bisulfite conversion of DNA using the EZ DNA methylation kit (Zymo Research, Orange, CA, USA), the methylation status was quantified using MethyLight assay in the following eight CIMP markers: *CACNA1G*, *CDKN2A (p16)*, *CRABP1*, *IGF2*, *MLH1*, *NEUROG1*, *RUNX3*, and *SOCS1*. The primer sequences and polymerase chain reaction (PCR) conditions have been described previously [29, 30]. *M.*SssI-treated genomic DNA was used as a reference sample. Percentage of methylated reference (PMR) at a particular locus was calculated by dividing the *GENE/ALU* ratio of a patient sample by the *GENE/ALU* ratio of the *M.*SssI-treated human genomic DNA sample and multiplying by 100. MethyLight assay was repeated in triplicate, and of the three measured values, the median was regarded as a representative value of methylation level of each marker. A CpG island locus with PMR >4 was considered to be methylated [4, 29, 31]. CIMP status was defined according to the number of methylated markers: CIMP-high (methylation at ≥5 markers), CIMP-low (1–4 markers), or CIMP-negative (0 marker) [4, 29, 31].

The microsatellite status of each tumor was determined by evaluating the five microsatellite markers (D2S123, D5S346, D17S250, BAT25, and BAT26). Either forward or reverse primer for each marker was labeled with fluorescence, and PCR products were electrophoresed and analyzed. We classified MSI status as follows: MSI-high (MSI-H; instability at two or more microsatellite markers), MSI-low (MSI-L; instability at one marker), or microsatellite stable (MSS) (no instability). Only MSI-H was regarded as having MSI, and MSI-L was grouped with MSS [4, 32].

Analysis of *KRAS* and *BRAF* mutation was performed as previously described [32]. DNA was extracted from paraffin-embedded tissue, and *KRAS* mutation (codon 12 and 13 of exon 2) was analyzed by using hemi-nested PCR method followed by direct sequencing. *BRAF* mutations at codon 600 (V600E) were analyzed by using a real-time PCR-based allelic discrimination method [32].

### Statistical analysis

The primary objective of this study was to investigate the effect of promoter methylation status on the treatment outcome (OS and DFS) and their association with sex in colorectal cancer patients treated with adjuvant FOLFOX chemotherapy. The clinical database was last updated in October 2014. DFS was calculated from the date of operation to the first date of documented recurrence or death. Data from patients who were free of recurrence were censored at the date of the last follow-up visit for DFS. In the analysis of OS, death from any cause was the primary end point. Categorical variables were compared using the chi-square test. OS and DFS were calculated using the Kaplan-Meier method, and comparisons were made using the log-rank tests. HR was calculated using the Cox proportional hazard model, and baseline characteristics were adjusted by using backward stepwise model including covariates which have the prognostic role: age (continuous variable), sex, stage (II vs*.* III), histology (mucinous adenocarcinoma vs*.* others), tumor location (proximal vs*.* distal), angiolymphatic invasion, venous invasion, perineural invasion, and MSI status. Two-sided *p* values of less than 0.05 were considered statistically significant. Statistical analysis was performed with SPSS software for Windows, version 18.0 (SPSS, Chicago, IL, USA).
